# Deletion of discoidin domain receptor 2 attenuates renal interstitial fibrosis in a murine unilateral ureteral obstruction model

**DOI:** 10.1080/0886022X.2019.1621759

**Published:** 2019-06-06

**Authors:** Xi’an Li, Xin Bu, Fei Yan, Fuli Wang, Di Wei, Jiarui Yuan, Wanxiang Zheng, Jin Su, Jianlin Yuan

**Affiliations:** a Department of Urology, Xijing Hospital, Fourth Military Medical University, Xi’an, Shaanxi, China;; b Department of Biochemistry and Molecular Biology, Fourth Military Medical University, Xi’an, Shaanxi, China;; c Department of Gene Technology, Fourth Military Medical University, Xi’an, Shaanxi, China;; d Department of Biochemistry, University of Washington, Seattle, WA, USA

**Keywords:** Discoidin domain receptor 2, renal interstitial fibrosis, unilateral ureteral obstruction, deletion mutation, calcium dobesilate

## Abstract

**Background:** Renal interstitial fibrosis is a common pathway of chronic kidney disease to end-stage renal disease, which is characterized by an imbalance between the synthesis and degradation of the collagen-rich extracellular matrix (ECM). While, discoidin domain receptor 2 (DDR2) can be activated when it binds to some types of collagen. Therefore, we hypothesized that DDR2 may be a major player in renal interstitial fibrosis.

**Methods:** Renal histologic analysis, real-time PCR analyses and hydroxyproline assay were performed in DDR2-deficient mice and wild-type mice after unilateral ureteral obstruction; C57 mice were randomly divided into sham operation group (Sham group, *n* = 4), renal interstitial fibrosis model group (UUO group, *n* = 4), and calcium dobesilate treatment group (CDT group, *n* = 4), preparation of renal interstitial fibrosis model by unilateral ureteral obstruction (UUO), CDT Group was treated with calcium dobesilate orally, Sham group and UUO group were given double distilled water, HE staining, Masson staining, real-time quantitative PCR were detected after 14 days of UUO in mice to observe the renal interstitial fibrosis degree.

**Results:** DDR2 expression was dramatically increased in the obstructed kidney; In contrast to wild-type mice that developed severe interstitial fibrosis, the DDR2-deficient mice displayed only moderate fibrotic changes; Compared with the UUO group, the degree of renal interstitial fibrosis in CDT group was relieved after operation 14 day.

**Conclusion:** DDR2 might play an important role in the development of RIF; Calcium dobesilate can affect the expression of DDR2 and improve the renal interstitial fibrosis in mice.

## Introduction

Renal interstitial fibrosis (RIF) is common in the progression of almost all chronic kidney diseases towards end-stage renal failure [1], and usually has a more severe impact on renal function than glomerular sclerosis [2]. An imbalance of collagen-based ECM synthesis and degradation is one of the main mechanisms of RIF [3]. Unilateral ureteral obstruction (UUO) is an ideal animal model of RIF [4], since it is characterized by progressive RIF and renal tubular atrophy [5].

Discoidin domain receptors (DDRs), encompassing DDR1 and DDR2, are unusual in that they become autophosphorylated in response to binding collagen, in contrast to receptor tyrosine kinases (RTKs) which are predominantly activated by soluble factors [6]. DDR2 is mainly expressed in mesenchymal cells [7], encompassing multiple cell types, such as smooth muscle cells, osteoblasts, and fibroblasts [8]. The mesenchymal DDR2 expression shows highest levels in skeletal muscle, skin, kidney, and lung tissue [9]. DDR2 was demonstrated to be associated with several pathological processes including hepatic fibrosis, osteoarthritis, wound healing, and tumor metastasis [10–13]. Nevertheless, the functional role of DDR2 in renal interstitial fibrosis remains unclear.

## Materials and methods

### Experimental animals

A heterozygous *slie* mouse colony with a deletion mutation of the DDR2 gene [14] was purchased from Jackson Laboratory (Bar Harbor, ME, USA) and intercrossed to generate homozygotes. Genotyping of all mice was performed by quantitative real-time polymerase chain reaction (qPCR) on an ABI 7500 instrument (Applied Biosystems, Foster City, CA, USA) using TaqMan probes (Takara, Kyoto, Japan), according to the manufacturer’s instructions. Mice aged 8–10 weeks and weighing 18 to 22 g were used for the experiments, encompassing DDR2-deficient mice and WT littermates. The animals were housed in pathogen-free conditions, placed on a regular diet and allowed free access to water. All animal procedures were in accordance with the Fourth Military Medical University of Animal Care and Use Committee.

### Unilateral ureteral obstruction

DDR2-deficient mice and WT littermates were anesthetized by an intraperitoneal injection of 1 mg/kg 1% pentobarbital sodium, and an incision was made in the left flank. The ureter was freed from the surrounding tissue, and UUO was performed by double-ligating the upper one-third of the left ureter using 4–0 silk [15].

### Renal histological analysis

After euthanizing the animals with an intraperitoneal injection of 1 mg/kg 1% pentobarbital sodium, the obstructed and contralateral kidneys were extracted, immersed for 24 h in 4% formalin, and embedded in paraffin after alcohol dehydration. Sections were stained with hematoxylin-eosin (Baso, China), Masson’s trichrome stain (Baso, China)

### Real-Time quantitative PCR

For the extraction of total RNA, both the obstructed and contralateral kidneys were harvested, and RNA was extracted using the Trizol reagent (Takara, Kyoto, Japan). RNA quality was tested by measuring the ratio of optical densities at 260 and 280 nm. We used the reverse transcription kit (Takara, Kyoto, Japan) to convert RNA into cDNA. cDNA was amplified by PCR on a Light Cycler 480 (Roche Diagnostics, Meylan, France) using SYBR Green (Fast Start DNA Master SYB-R Green I; Roche Applied Science, Roche Diagnostics). Specific primers for alpha smooth muscle actin (α-SMA), DDR2, fibronectin, collagen1 α1 chain (COL1A1), HO-1, and glyceraldehyde-3-phosphate dehydrogenase (GAPDH) (Table 1) were designed by Sangon Biotech (Shanghai, China). Amplification was conducted using the following PCR conditions: 95 °C for 2 min, followed by 40 cycles at 95 °C for 5 s and 60 °C for 30 s, 95 °C for 15 s, 60 °C for 30 s, and 95 °C for 15 s. To normalize the real-time quantitative PCR (qPCR) results, we used Bio-Rad CFX-Manager 3.0 software (Bio-Rad, USA), and the results were expressed as 2-△Cq, where Cq is the cycle threshold number. Dissociation curves were analyzed after each run for each amplicon to assess the specificity of the quantification when using SYBR Green.

**Table 1.** Primers used for real-time qPCR.

mRNA	Strand	Sequence
GAPDH	Sense	5′-CCTTCCGTGTTCCTACCC-3′
	Antisense	5′-GGAGTTGCTGTTGAAGTCG-3′
a-SMA	Sense	5′-GACGCTGAAGTATCCGATAGAACACG-3′
	Antisense	5′-CACCATCTCCAGAGTCCAGCACAAT-3′
DDR2	Sense	5′-AAGACGGAGTTGGATCTGGA-3′
	Antisense	5′-AATAATTGAGGAGGAGCGGG-3′
Fibronectin	Sense	5′-TCTGGGAAATGGAAAAGGGGAATGG-3′
	Antisense	5′-CACTGAAGCAGGTTTCCTCGGTTGT-3′
COL1A1	Sense	5′-GGAGGGCGAGTGCTGTGCTTT-3′
	Antisense	5′-GGGACCAGGAGGACCAGGAAGT-3′
HO-1	Sense	5′-CTGTGTAACCTCTGCTGTTCC-3′
	Antisense	5′-CCACACTACCTGAGTCTACC-3′

### Hydroxyproline assay

We used the hydroxyproline assay kit (Jiancheng Bioengineering Institute, Nanjing), according to the manufacturer’s protocol. Briefly, accurately weighed wet kidney tissue was hydrolyzed and mixed carefully. The resulting tissue samples were incubated at 95 °C in a water bath for 20 min. After cooling, the indicator was added turning the mixture red, after which acetate was added dropwise with a 200 µL pipette to adjust the pH until the mixture turned yellow-green. Subsequently, distilled water was added under constant mixing to a total volume of 10 mL, 4 mL of which were drawn and combined with an appropriate amount of activated carbon. After centrifugation, 1 mL of the supernatant was mixed with the specified reagents and incubated for the specified times according to the kit’s instructions, after which the mixture was centrifuged and the absorbance of the supernatant at a wavelength of 550 nm was measured.

### Western blotting

Tissues were lysed with radioimmunoprecipitation assay (RIPA) buffer supplemented with protease inhibitors (Roche, Branchburg, NJ, USA). Protein lysates were subjected to 10% SDS-PAGE, transferred to nitrocellulose membrane (Bio-Rad, Hercules, CA, USA), and incubated with the indicated antibody: Goat anti-mouse monoclonal anti-β-actin antibody (Sigma Aldrich, St. Louis, MO, USA), rabbit anti-mouse Fibronectin antibody (Biosynthesis Biotechnology, Beijing, China), rabbit anti-mouse COL1A1 (Boster, Wuhan, China). Bands were developed with enhanced chemiluminescence (ECL) system (Amersham Bioscience, Buckinghamshire, UK).

### Calcium dobesilate assay

C57 mice were randomly divided into sham operation group (Sham group, *n* = 4), renal interstitial fibrosis model group (UUO group, *n* = 4) and calcium dobesilate treatment group (CDT group, *n* = 4), preparation of renal interstitial fibrosis model by unilateral ureteral obstruction (UUO), CDT Group was treated with calcium dobesilate orally 150 mg·kg^−1^·d^−1^ (calcium dobesilate, purchased from Xi’an Lijun Pharmaceutical Co., Ltd., Xi `an, China. Product lot number: 15041–27, 10 mg/mL^−1^ solution prepared by adding double steamed water), Sham group and UUO group were given equal volume of double distilled water, HE staining, Masson staining, and real-time quantitative PCR was detected after 14 days of UUO.

### Statistical analysis

All experiments were performed at least three times independently. We use analysis of variance between the two groups or more than two groups followed by Independent Student *t*-test or one-way ANOVA. Values of *p* < .05 were considered to indicate statistical significance. All data are expressed as means ± SD from at least three times independent experiments.

## Results

### UUO caused obvious renal interstitial fibrosis

**Figure 1.**
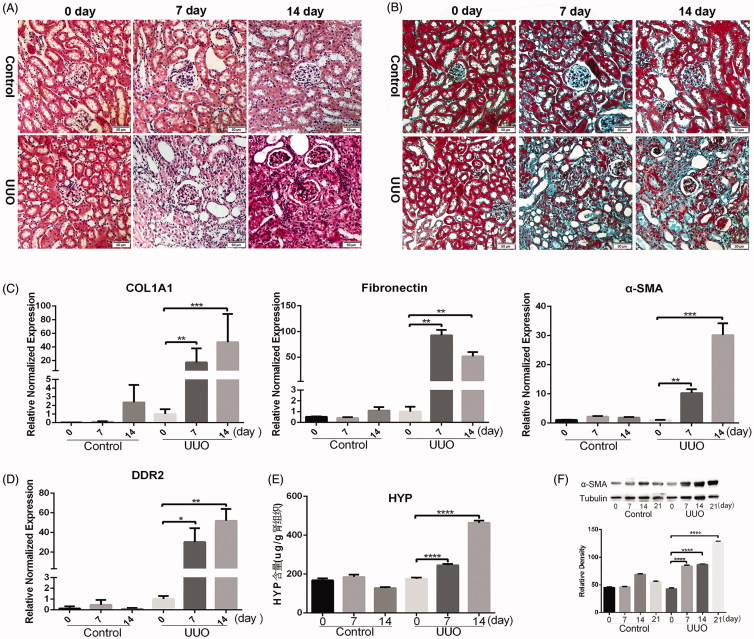
UUO caused obvious renal interstitial fibrosis. Pathological changes in the kidney at 0, 7, and 14 days after the UUO operation (A) HE staining (scale bar = 50−µm). (B) Masson staining (scale bar = 50 µm). (C) The relative gene expression of COL1A1, α-SMA and fibronectin in the kidney at 0, 7, and 14 days after the UUO operation. (D) Relative normalized gene expression of DDR2 in the kidney, evaluated by real-time qPCR at 0, 7, and 14 days after the UUO operation. (E) The hydroxyproline content in the kidney at 0, 7, and 14 days after the UUO operation (****p < .0001). (F) Western blot analysis of the protein level of α-SMA at 0, 7, 14, and 21 days after the UUO operation.

Upon standard optical microscopy, HE-stained mouse kidney tissue showed normal glomerular and renal tubular and interstitial structures in the control group. By contrast, at 7 and 14 days after UUO, part of renal tubular epithelial cells progressively degenerated, swelling and renal tubular atrophy appeared (Figure 1(A)). Masson tissue staining confirmed that the control group had no obvious renal interstitial collagen fibers, while the UUO group showed increasing amounts of blue-stained interstitial collagen fibers, indicating progressively more serious renal interstitial fibrosis after unilateral ureteral ligation, especially after 14 days post-UUO treatment (Figure 1(B)). Real-time quantitative PCR analysis of the relative expression of COL1A1, fibronectin, and α-SMA showed that the amounts increased significantly in the renal tissue of UUO mice compared with the control group. Moreover, the expression of COL1A1 and α-SMA increased gradually at 7 and 14 days after unilateral ureteral ligation. By contrast, fibronectin expression first increased gradually, but decreased slightly after fourteenth days (Figure 1(C)). The results of real-time PCR showed that the relative expression of the DDR2 gene in kidney tissue gradually increased at 7 and 14 days after the UUO operation in wild-type mice (Figure 1(D)). Hydroxyproline is found almost exclusively in collagen, and its content can indirectly reflect the content of collagen. The results of hydroxyproline determination showed a significantly higher content in UUO mice after 7 and 14 days than in the control group, with a gradually increasing trend over 14 days (Figure 1(E)). Western blot analysis of the protein level of DDR2 and α-SMA showed that the protein level of α-SMA increased gradually at 7, 14, and 21 days after UUO (Figure 1(F)).

### Deletion of discoidin domain receptor 2 attenuates renal interstitial fibrosis

**Figure 2.**
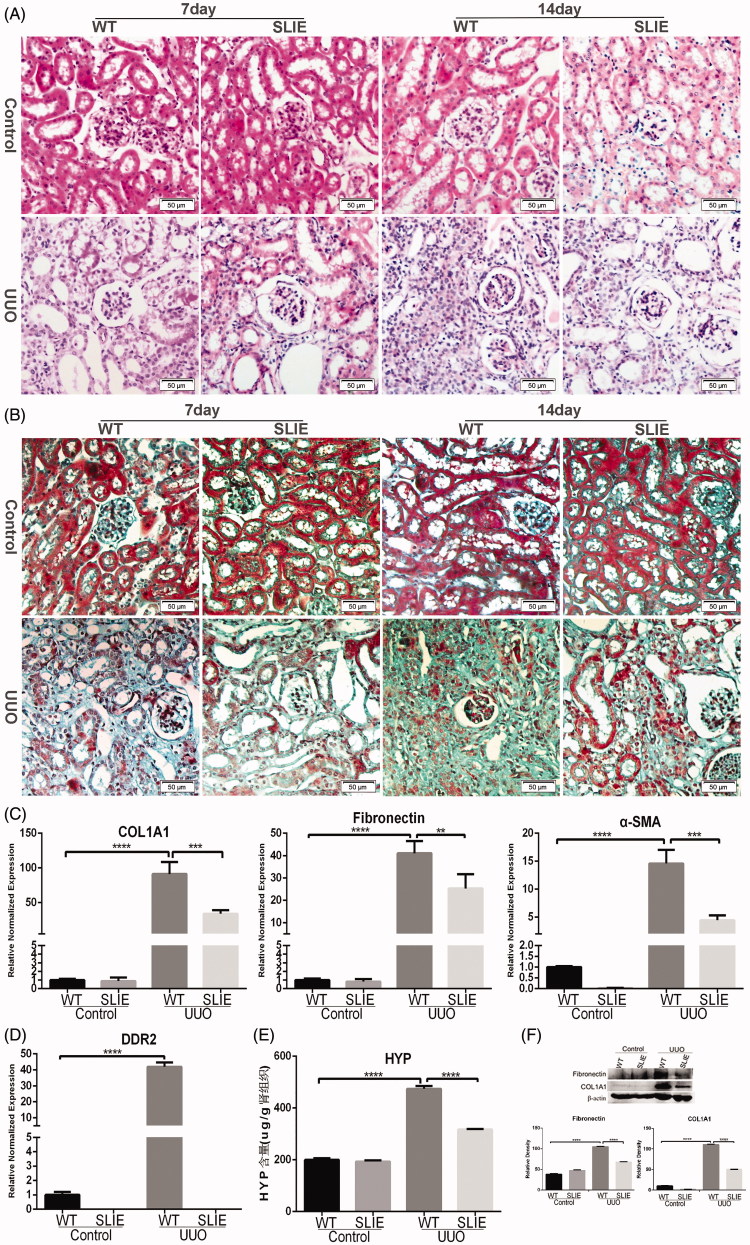
Deletion of discoidin domain receptor 2 attenuates renal interstitial fibrosis. (A) HE staining (scale bar = 50 µm). (B) Masson staining (scale bar = 50 µm). (C) Relative normalized gene expression of COL1A1, fibronectin and α-SMA, evaluated by real-time qPCR at 14 days after the UUO operation (**p < .01, ***p < .001, ****p < .0001). (D) Relative normalized gene expression of DDR2, evaluated by real-time qPCR at 14 days after the UUO operation (****p < .0001). (E) The content of hydroxyproline in the kidney at 14 days after the UUO operation (****p < .0001). (F) Western blot analysis of the protein level of fibronectin and COL1A1 14 days after UUO in the DDR2-deficient mice and the wild-type mice.

The results of HE-staining showed that the glomerular, tubular and interstitial structures in the control kidneys were normal. The renal tubules of wild-type mice were atrophied, reduced, after UUO treatment. Compared with wild-type mice, the pathological changes of DDR2-deficient mice were significantly reduced, and there were different degrees of swelling and degeneration of renal tubular epithelial cells. Unsurprisingly, the pathological changes 14 days after UUO were more severe than after 7 days in both wild-type and DDR2-deficient mice (Figure 2(A)). The results of Masson staining showed that the renal interstitium had no obvious blue-stained collagen fibers in the control kidneys, while there were large amounts of collagen fibers in the renal interstitium of UUO-treated wild-type mice. By contrast, the renal interstitial collagen fibers of DDR2-deficient mice were significantly reduced compared to those of wild-type mice, and the changes 14 days after UUO were more pronounced than after 7 days (Figure 2(B)). Real-time quantitative PCR showed that the expression of the fibrosis-associated genes COL1A1, fibronectin and α-SMA in the kidneys of DDR2-deficient mice was decreased compared with those of wild-type mice (Figure 2(C)). Moreover, the relative normalized expression of DDR2 in kidneys of wild-type mice was significantly increased 14 days after UUO (Figure 2(D)). Similarly, the content of hydroxyproline in the kidneys of wild-type mice was gradually increased 7 and 14 days after UUO, while its concentration in the DDR2-deficient mice was decreased significantly compared with wild-type mice 14 days after UUO (Figure 2(E)). Western blot analysis of the protein level of fibronectin and COL1A1 showed that the protein level of fibronectin and COL1A1 reduced at 14 days after UUO in the DDR2-deficient mice compared with the wild-type mice (Figure 2(F)).

### Calcium dobesilate affected the expression of DDR2 in the renal tissue of UUO mice, and reduced the renal interstitial fibrosis

**Figure 3.**
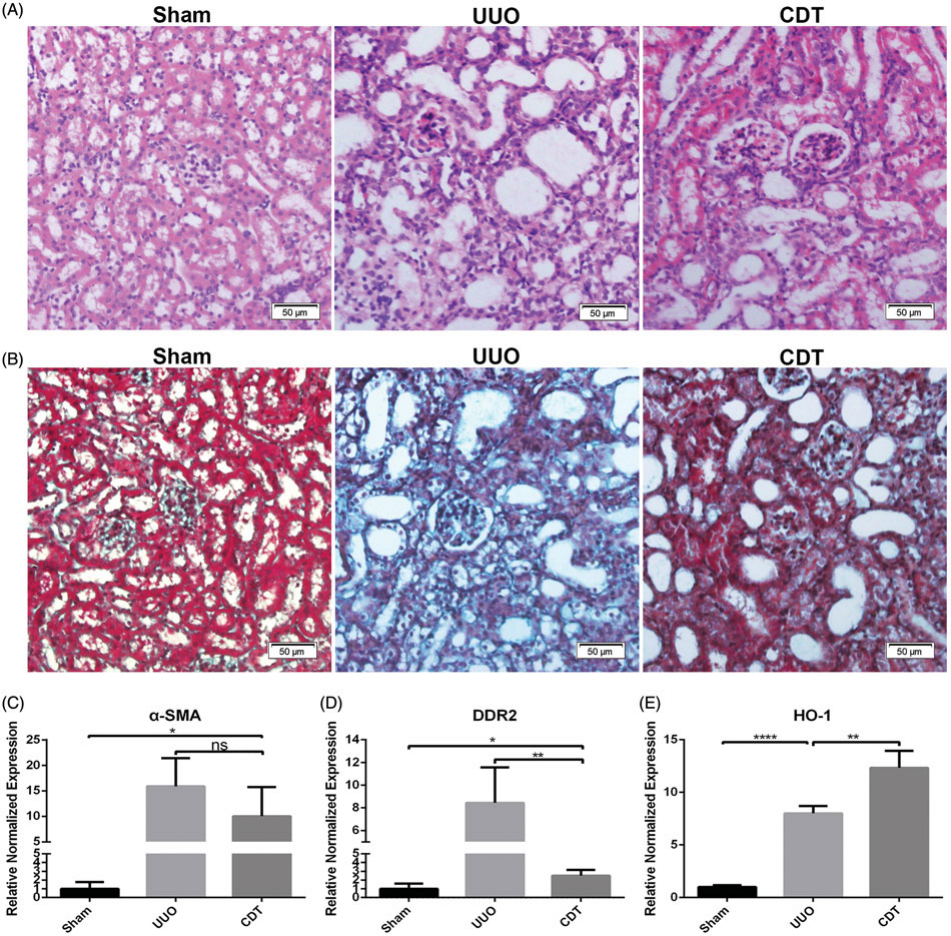
Calcium dobesilate affected the expression of DDR2 in the renal tissue of UUO mice, reduced renal interstitial fibrosis, and protected the renal function. Pathological changes in the kidney at 14 days after the UUO operation. (A) HE staining (scale bar = 50 µm). (B) Masson staining (scale bar = 50 µm). (C) The relative expression of the fibrosis related gene α-SMA in the kidney at 14 days after the UUO operation (*p < .05). (D) The relative gene expression of DDR2 in the kidney at 14 days after the UUO operation (*p < .05, **p < .005). (E) The relative expression of the renoprotective gene HO-1 in the kidney at 14 days after the UUO operation (**p < .005, ****p < .0001).

HE staining of renal tissues of the mice showed that the renal structure in the sham intervention group was basically normal, while in the UUO group renal tubular atrophy was present. Notably, compared to the non-treated UUO group, the calcium dobesilate (CDT)-treated group had significantly reduced pathological changes, including swelling and vacuolar degeneration of renal tubular epithelial cells of different degrees (Figure 3(A)). Masson staining revealed that the sham group had renal interstitium without obvious blue-stained collagen fibers, while the UUO group presented with large amounts of blue-stained interstitial collagen fibers. Furthermore, the CDT group had significantly reduced amounts of renal interstitial collagen fibers compared with the UUO group (Figure 3(B)). To corroborate these results, real-time PCR analysis of α-SMA, DDR2 and the protective gene of renal function heme oxygenase-1 (HO-1) expression in kidney tissues was performed 14 days after UUO surgery. The results showed that the expression of DDR2 and HO-1 increased significantly in renal tissue of UUO mice compared with the sham group. Furthermore, the expression of DDR2 and α-SMA in renal tissues of CDT mice were reduced (Figure 3(C,D)), and the expression of HO-1 in renal tissues of CDT-treated mice was significantly increased compared to the non-treated UUO group (Figure 3(E)).

These results show that DDR2-deficient mice had a significantly reduced degree of renal interstitial fibrosis following UUO operation.

## Discussion

Renal interstitial fibrosis is one of the main pathological features of renal disease progressing towards end-stage renal failure. At present, there are no good clinical solutions. UUO is an experimental intervention that can cause renal interstitial fibrosis in a short period of time and with uniform lesions, due to its similarity to pathological processes found in clinical obstructive diseases [16]. Its damage is mainly mediated by renal interstitial fibrosis, without causing significant glomerular lesions [17]. It is consequently widely used to study renal interstitial fibrosis [18].

DDR2 is a collagen-binding member of the receptor tyrosine kinase family [19]. It is a transmembrane protein composed of intracellular, transmembrane and extracellular parts [20], with an extracellular discoidin domain [21]. Studies have shown that mice with a DDR2 deletion had significantly reduced bleomycin-induced pulmonary fibrosis. Conversely, upregulation of DDR2 expression significantly increased bleomycin-induced pulmonary fibrosis in mice. At the same time, cytological analysis showed that DDR2 can promote the activation of lung fibroblasts [22]. Similarly, George et al. implicated DDR2 as a key regulator in Ang II-induced cardiac fibrosis. The authors revealed a positive interrelationship between DDR2 and collagen production, which was dependent on the activation of the NF-κB signaling pathway [23]. Seon Ju Yeo et al. showed that a genetic deletion of the collagen receptor DDR2 in mice led to slower deposition of fibrillar collagen by cardiac fibroblasts [24]. Xi-Hong Zhang et al. showed that the expression of DDR2 is enhanced in a rat model of alcohol-induced liver fibrosis. Moreover, the expression of DDR2 is closely associated with collagen in the fibrotic liver tissues [25]. However, another study reported that the loss of DDR2 promotes hepatic fibrosis after chronic carbon tetrachloride exposure, acting through altered paracrine interactions between hepatic stellate cells and liver-associated macrophages [11]. We applied HE and Masson staining, real-time quantitative PCR, and the hydroxyproline assay to investigate the fibrotic changes in the kidneys of UUO-treated wild-type and DDR2-deficient mice. All results indicated significantly reduced fibrosis in the DDR2-deficient mice compared with wild-type littermates. This was consistent with the results reported by Guerrot et al., which showed that DDR1-null mice are protected against interstitial fibrosis induced by UUO [15]. We speculate that the possible mechanism is that DDR2 may impact the collagen production. Overall, our work demonstrates that deletion of DDR2 attenuates renal interstitial fibrosis. Thus, DDR2 may be a major player in renal interstitial fibrosis, indicating its promising therapeutic potential.

There is no ideal drug for the treatment of renal interstitial fibrosis. Takahiro Uchida et al. showed that the various protective effects of dipeptidyl peptidase 4 inhibition in nondiabetic mice with UUO [26]. Jin Liang et al. suggest that resveratrol treatment inhibits oxidative stress, Smad3 acetylation, and renal interstitial fibrosis [27]. And Cui Zhang et al. showed that resveratrol can reduce the expression of eIF2a and ATF4 protein and subsequently prevents the excessive apoptosis of renal tubular epithelial cells and delays the renal fibrosis development [28]. Calcium dobesilate can reduce the permeability of capillary, inhibit platelet aggregation, and reduce the blood viscosity. Calcium dobesilate is mainly used in treatment of microvascular disease in clinical, which is widely used in varicose veins, diabetic retinopathy, diabetic nephropathy and other disease treatment. Our research shows that Calcium dobesilate can affect the expression of DDR2 and improve the renal interstitial fibrosis in mice. which provides a theoretical basis for delaying the progression of renal interstitial fibrosis. However, the mechanism is not clear. Our ongoing research is focusing on the regulative mechanism of calcium dobesilate.

## Conclusion

In conclusion, unilateral ureteral obstruction model in mice could induce renal interstitial fibrosis. The expression of DDR2 increased gradually in the progression of renal interstitial fibrosis in mice, and the renal interstitial fibrosis of DDR2-deficient mice alleviated significantly than that in wild-type mice. Calcium dobesilate can affect the expression of DDR2 and improve the renal interstitial fibrosis in mice.
